# Imaging Flow Cytometry in HIV Infection Research: Advantages and Opportunities

**DOI:** 10.3390/mps8010014

**Published:** 2025-02-03

**Authors:** Kirill. A. Elfimov, Dmitriy. A. Baboshko, Natalya. M. Gashnikova

**Affiliations:** State Research Center of Virology and Biotechnology “Vector”, Retrovirus Department, Koltsovo 630559, Russia; d.baboshko@g.nsu.ru (D.A.B.); nmgashnikova@gmail.com (N.M.G.)

**Keywords:** HIV, imaging flow cytometry, virology, viral reservoirs

## Abstract

The human immunodeficiency virus (HIV) is a type of retrovirus that infects humans and belongs to the Lentivirus group. Despite the availability of effective treatments, HIV infections are still increasing in some parts of the world, according to the World Health Organization (WHO). Another major challenge is the growing problem of HIV becoming resistant to drugs. This highlights the importance of ongoing research to better understand HIV and find new ways to stop the virus from spreading in the body. Scientists use a variety of methods to study HIV, including techniques from molecular and cellular biology. Many of these methods rely on fluorescent dyes to help visualize specific parts of the virus or infected cells. This article focuses on a technique called imaging flow cytometry, which is particularly useful for studying HIV. Imaging flow cytometry is unique because it not only measures fluorescence (light emitted by the dyes) but also captures images of each cell being analyzed. This allows researchers to see where the fluorescence is located within the cell and to study the cell’s shape and structure in detail. Additionally, this method can be combined with machine learning to analyze large amounts of data more efficiently.

## 1. Introduction

The human immunodeficiency virus (HIV) is a type of retrovirus belonging to the Lentivirus group that can infect humans. If left untreated, HIV can lead to acquired immunodeficiency syndrome (AIDS). HIV is particularly complex because it integrates itself into the human genome, making it difficult to eliminate. It can also hide in the body for long periods, creating “reservoirs” where the virus remains inactive but can reactivate later. Additionally, HIV damages immune cells, making it harder for the body to fight infections [1].

As of 22 July 2024, around 39.9 million people worldwide are living with HIV, and 30.6 million of them are receiving antiretroviral therapy (ART) [2]. ART has proven to be highly effective—not just for managing HIV in individuals but also for helping to control and reduce the spread of the virus in communities [3]. However, while ART can keep the virus under control, it does not cure HIV, meaning patients need to take these medications for life.

As more people receive ART, the problem of HIV drug resistance is becoming more important [4,5]. Even the newest and most effective drugs, like lenacapavir, can stop working if the virus becomes resistant to them. This is why these drugs are usually given alongside other treatments [6]. To address this challenge, it is crucial to study how HIV works and find new ways to stop the virus from multiplying or to completely remove it from the body. To do this, scientists need reliable lab models of HIV infection. When using these models, researchers often study large groups of cells using a technique called flow cytometry, which provides highly accurate and detailed data.

Flow cytometry, a powerful tool for analyzing cells, was first used to study HIV infection back in 1986 [7,8]. In the beginning, it was mainly used for basic clinical tests, such as counting specific types of immune cells called CD4+ and CD8+ T lymphocytes. Over time, advances in antibody development, dyes, and equipment have made flow cytometry even more effective and versatile.

Flow cytometry is a powerful tool for analyzing cells, and it stands out because it uses fluorescent dyes (called fluorochromes) more effectively than any other method. Modern flow cytometry systems can measure up to 19 or more different characteristics from just 1 sample [9]. Thanks to advanced, highly sensitive equipment, it can even detect tiny particles, like virions, that are at the very edge of what light microscopes can see [10]. One of the latest breakthroughs in this field is imaging flow cytometry, which combines traditional flow cytometry with imaging capabilities. This technology is now available in several commercial systems.

The first category includes devices that are more like advanced cell counters than traditional flow cytometers. These systems capture images of cells using fluorescence imaging, with the cells placed on a solid surface, such as glass or a plate. One example of this technology is the Celigo™ image cytometer (Revvity, Inc., Lawrence, The State of Kansas, USA). This device automatically takes pictures of cells located in the wells of a plate. It can focus either manually or automatically, with the automatic mode relying on contrast detection. Celigo™ uses LED lights for illumination and can capture images in brightfield as well as in four fluorescence channels (blue, green, red, and far red), each equipped with specific filters. The Celigo™ image cytometer has been used in important research. For example, it was used to study therapeutic antibodies for treating MERS-CoV. In this study, the cytometer measured how effectively the antibodies bound to the MERS-CoV S protein and interacted with cell receptors [11]. In another study focused on finding potential drugs against SARS-CoV-2, Celigo™ was used to track cell survival and viral replication [12].

This method has several advantages, including fast and simple analysis, as well as lower equipment costs compared to traditional flow cytometers. However, it also has some drawbacks. For example, it cannot measure differences within cells (intracellular heterogeneity) and is less sensitive than flow cytometers that use visualization techniques.

The second type of visualization tool is called high-content imaging (HCI). HCI is an advanced imaging method that allows scientists to capture and analyze multiple details about cells and biological samples at the same time. This technique involves three main steps: image acquisition (taking pictures), image processing (preparing the images for analysis), and image analysis (extracting useful information from the images) [13].

One of the key advantages of HCI is that it automates the entire process of collecting and analyzing data. This is made possible with the help of artificial intelligence (AI) and machine learning (ML), which can identify important biological patterns in the data without human bias [14].

HCI is a powerful tool that helps researchers gather detailed information from images of cells. The process begins with segmentation, where specialized software identifies and outlines individual cells or their parts in the images [15]. Once the cells are segmented, the software measures important features like cell size, shape, and the location of fluorescent markers. These measurements give researchers valuable insights into how cells behave and interact, helping them understand how cells respond to different treatments or conditions. For example, researchers used the CELENA X imaging system (CELENA^®^ X High Content Imaging System, Logos Biosystems, ©Aligned Genetics, Inc., Anyang, Gyeonggi-do, Republic of Korea) to study how SARS-CoV-2 persists in alveolar macrophages. They discovered that this process is closely linked to the activity of NK cells, which produce a protein called IFN-Y [16]. In their study, they used the CELENA X platform and its software to analyze fluorescent images. They specifically looked at the expression of several proteins, including HLA-E, NKG2A, NKG2C, and NKG2E (which activate NK cells), as well as CD107a (a cytotoxic T lymphocyte degranulation marker).

**Figure 1:**
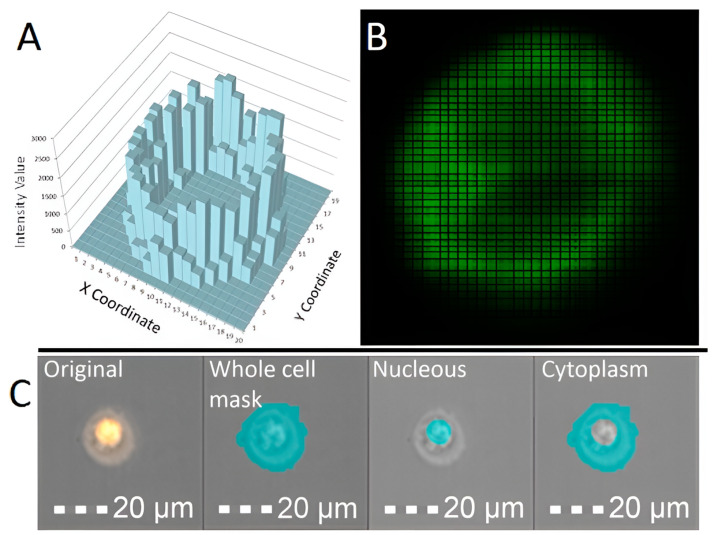
This figure shows how Cytek^®^ Amnis^®^ imaging flow cytometers create images and masks. (**A**): The CCD camera collects signals from individual pixels. The Z-axis represents the brightness of the fluorescent signal, while the X and Y axes show the cell’s position on a flat surface. Each small square (pixel) on the X and Y grid captures part of the fluorescent signal. (**B**): A 2D image is formed based on the signals from the CCD camera. The brightness of each part of the image depends on how many photons (light particles) come from different areas of the cell. (**C**): This is an example of creating a new mask by combining basic masks using the logical NOT operator.

The third category includes imaging flow cytometers, which are designed similarly to traditional flow cytometers. These devices use lenses with magnifications between 20× and 60×, along with special CCD cameras that use time-delay integration (TDI) technology to capture images. The camera “tracks” cells as they move through its field of view, collecting fluorescent signals—similar to how exposure works in fluorescence microscopy (Figure 1A). To create images, the camera records the number of photons coming from different parts of a cell (Figure 1B). Because the flow rate is slow, cells stay in the camera’s view long enough to gather enough fluorescent signals, resulting in high-resolution and clear images.

To reduce noise in specific fluorescence channels, a special tool called a spectral compensation matrix is used. This matrix is created using samples with a single fluorescent dye (compensation control). The software analyzes each image using 86 different parameters. These include standard flow cytometry measurements, like side scatter and intensity, as well as many other features related to the size, shape, location, comparison, and texture of objects. To focus on specific parts of the image, such as cell membranes, nuclei, or areas where cells interact, the software uses specialized masks and combinations of these masks (Figure 1C). By combining 86 parameters, 22 masks, and 12 fluorescence channels, researchers can identify different groups of cells based on a wide range of features. A key feature of this system is the ability to create custom mask combinations using logical operators like AND, OR, and NOT (Figure 1C). The software also allows researchers to analyze each parameter for individual objects, which greatly improves the statistical accuracy of the study. Without this technology, analyzing this much data using traditional microscopy would take weeks and would be nearly impossible for rare events (less than 1 in 10,000).

Machine learning and deep learning are powerful tools in imaging flow cytometry. These methods can make data analysis faster, easier, and even uncover new biological insights. However, machine learning models perform best when working with images that have consistent conditions, such as lighting, object position, and shadows [17]. Fortunately, imaging flow cytometry naturally meets these requirements, as it captures images of cells under controlled and uniform settings.

Convolutional neural networks (CNNs) are a popular tool for analyzing images. They work by using convolution, where a filter moves across the image to detect small details like edges, shapes, and textures. After this step, a function called Rectified Linear Unit (ReLU) is applied. ReLU helps by removing negative values while keeping positive ones, which makes it easier for the network to learn complex patterns in the data. This improves the efficiency and reliability of the learning process and helps create better representations of the data [18]. Finally, the results are “pooled,” which reduces the size of the data but keeps the most important features intact.

These operations happen in layers, one after another. Each layer extracts more complex features from the data, building on what the previous layer found. After the convolutional layers, the data are turned into a simpler form using fully connected layers. These layers take high-dimensional input data (like a 60 × 128 pixel image, captured with a 40× Cytek^®^ Amnis^®^ ImageStream^®^X Mk II lens) and reduce it to a smaller, more manageable size (like a 1 × 1 cell–matrix).

Machine learning is widely applied to analyze data from imaging flow cytometry. Researchers often recommend using specialized software, such as Cell-Profiler or CellProfiler Analyst, to distinguish cell populations with subtle morphological differences [19]. For example, H. Hennig et al. developed a method to classify Jurkat cells by their cell cycle stages (interphase, prophase, metaphase, anaphase, and telophase) without using labels. They achieved this by applying machine learning algorithms, such as Gradient Boosting and Random Forest, to analyze morphological features like granularity, cell area, brightness in light-field images, and other parameters [19].

In a separate study, Mariam Nassar et al. used imaging flow cytometry to categorize white blood cells into groups (lymphocytes, monocytes, eosinophils, and neutrophils) without the need for staining. The researchers focused on cell size, shape, and proportions to differentiate the cells. They employed various machine learning techniques, including Support Vector Machines, Random Forest, k-Nearest Neighbors (k-NN), and convolutional neural Networks (CNNs), to process and analyze the data [20].

In modern HIV research, scientists are shifting their focus from studying all types of cells in blood samples to analyze specific groups of cells. Two cell types are especially important: memory T cells, which often act as hidden reservoirs for HIV, and astrocytes, which are brain cells that support neurons and are involved in HIV replication in the central nervous system (CNS). Flow cytometry is a powerful tool for studying these cell populations because it can analyze tens of thousands of cells quickly. However, this method has some limitations. Without imaging, it is hard to confirm where specific signals come from, determine the exact location of fluorescent markers, or study rare events. To address these issues, microscopy is often used alongside flow cytometry. Imaging flow cytometry combines the strengths of both techniques—it works like a flow cytometer but also captures detailed images of cells. This review explores how imaging flow cytometry is being used in HIV research, particularly in studies where analyzing large numbers of cells is crucial, and highlights the advantages it offers over traditional methods.

## 2. The Latent HIV Reservoir Evaluation

Latent HIV reservoirs are inactive forms of the virus that remain hidden in the body. These reservoirs can become active and produce new viruses if ART is stopped or if the ARV levels in the body drop [1]. These hidden reservoirs are the main reason why HIV cannot yet be completely cured [10,21]. However, researchers are exploring several promising strategies to address this challenge.

One of the most discussed approaches is gene therapy, which has shown success in treating other diseases, such as spinal muscular atrophy in children (e.g., the drug Zolgensma) [22]. Despite this progress, there are currently no approved gene therapy treatments for infectious diseases like HIV [23]. However, some vaccines developed using gene therapy technology were approved [24]. For gene editing tools like CRISPR-Cas to work effectively against HIV, they need to be delivered directly into infected cells. This process faces challenges, such as identifying how many cells harbor the virus, ensuring the stability of these viral reservoirs, and making sure the CRISPR-Cas components can access the chromatin within the cells.

## 3. Cellular Reservoirs

HIV primarily targets CD4+ T-lymphocytes. Some of these cells can survive for a long time, which helps the virus create a stable and hidden reservoir in the body. When ART is paused, memory T-lymphocytes become the main source of new HIV particles. Memory T-lymphocytes are divided into three main types: central memory T cells (T_CM_), transitional memory T cells (T_TM_), and effector memory T cells (T_EM_) [25]. Additionally, recent research has identified immature memory T cells (T_SCM_) as another potential reservoir for HIV [26]. While there is ongoing debate about which type of memory T cell is most important for maintaining the viral reservoir, all of these cell types can carry HIV DNA. As a result, they are a key focus of research, particularly in studies using flow cytometry to analyze these cells.

Another important reservoir of HIV in the body involves tissue-resident macrophages, which are immune cells found in various tissues, such as bones, lungs, and the central nervous system [27]. These cells become infected during their early stage as monocytes in the bloodstream. Later, they migrate into different tissues, making it difficult to identify a specific location for this viral reservoir. While macrophages generally have a short lifespan, averaging around 90 days, some types, like microglia in the CNS, can survive for 2–4 years [28]. Despite their limited lifespan, macrophages play a significant role in spreading HIV. They can release free viral particles and transmit the virus directly to other cells, including newly arrived macrophages in tissues. The exact mechanisms of cell-to-cell HIV transmission during ART are not fully understood, but evidence suggests it does occur [29]. For example, certain antiretroviral drugs, such as tenofovir, the nucleoside reverse transcriptase inhibitor (NRTI), and efavirenz, the non-nucleoside reverse transcriptase inhibitor (NNRTI), are highly effective at blocking HIV replication in most cases. However, their ability to prevent infection is reduced when the virus spreads through direct cell-to-cell contact, such as via virological synapses [30]. This creates a cycle of infection: infected monocytes move from the bloodstream into tissues, transform into macrophages, and then infect other macrophages, sustaining the viral reservoir. The stability of this reservoir and the diversity of HIV variants within it remain unclear. However, it is important to note that macrophage reservoirs of HIV can potentially exist in almost any tissue at any time.

One of the most surprising places where HIV can hide is in a type of brain cell called astrocytes [31]. These cells are part of CNS and play a key role in the blood–brain barrier, which protects the brain and supports the health of neurons. Research has shown that astrocytes can become infected with HIV and may help the virus spread from cell to cell [32]. While it is still unclear whether astrocytes can produce new HIV particles, some studies suggest they might have this ability [33]. Additionally, astrocytes are long-lasting cells that can store HIV in a dormant state for a long time [34]. This makes the CNS a significant source of HIV particles in the blood when ART is stopped [35].

The latent reservoir of HIV can be detected using techniques such as fluorescent in situ hybridization (FISH) [10,21,36,37,38,39,40]. This method relies on DNA fragments called probes, which bind to specific target DNA sequences due to their complementary structure. FISH is commonly used for diagnosing viral infections, including hepatitis B and animal viruses [41,42], studying viral replication [43], analyzing the movement and location of viral genetic material within cells [44], and investigating HIV reservoirs. Although FISH may not be as precise as DNA sequencing, it is highly effective in determining the location and size of hidden viral reservoirs [45].

FISH staining has several important applications in HIV research. It helps measure the number of HIV proviruses (inactive viral DNA) in the cellular genome and assess the likelihood of HIV integration into different cell types, such as lymphocytes, sperm cells, and astrocytes. This technique is also useful for the highly sensitive detection of HIV in patients with conflicting diagnostic test results. Additionally, FISH can be combined with immunocytochemical staining to visualize modified histones, which can reveal the level of gene activity, including in areas where HIV proviruses have integrated into the genome [1].

HIV transcripts are important indicators of how HIV infection progresses within a cell. RNA-FISH is used to measure the activity of the HIV provirus [46], study how cells respond to treatments (such as detecting HIV RNA and Gag gene products) [47], and analyze the types and amounts of mRNA needed for different HIV behaviors (like rapid/high or slow/low replication). In addition to detecting HIV transcripts, RNA-FISH can also label RNA in host cells. This helps researchers study how cells respond to the virus early in infection and how they change their metabolism to produce viral proteins. These changes are seen in the amounts and types of RNA produced by host genes (such as PD-1, CTLA-4, CD160, and TIGIT) [48,49,50]. Like DNA detection, combining flow cytometry with imaging is a powerful method for RNA-FISH when the precise measurement of specific RNA molecules is needed.

There are many methods and protocols available for detecting HIV DNA and RNA using DNA-FISH [38,39,51,52] and RNA-FISH [47,53,54]. However, traditional flow cytometry has significant limitations. It can only provide semi-quantitative measurements of HIV provirus by detecting fluorescence, but it cannot determine where the signal is located (e.g., in the nucleus or cytoplasm) or confirm whether the probe has specifically bound to its target. In contrast, imaging flow cytometry offers a more complete solution. It allows for both visualization of the sample and precise counting of hybridization spots, making it possible to perform accurate quantitative measurements.

Latent HIV reservoirs can be identified using immunocytochemical analysis. One method involves detecting activated T cells by measuring the levels of specific signaling molecules, such as CD38 and HLA-DR, using a technique called spot counting [55]. In this study, researchers employed a scoring function to identify internalized HIV particles. They used Amnis flow cytometry to visualize how monocytes take up fluorescently labeled HIV-1 virions (HIV-1BaL-Tomato). To enhance the internalization process, the cells were treated with various antibodies. Afterward, images of the cells were captured using a flow cytometer [56].

**Figure 2:**
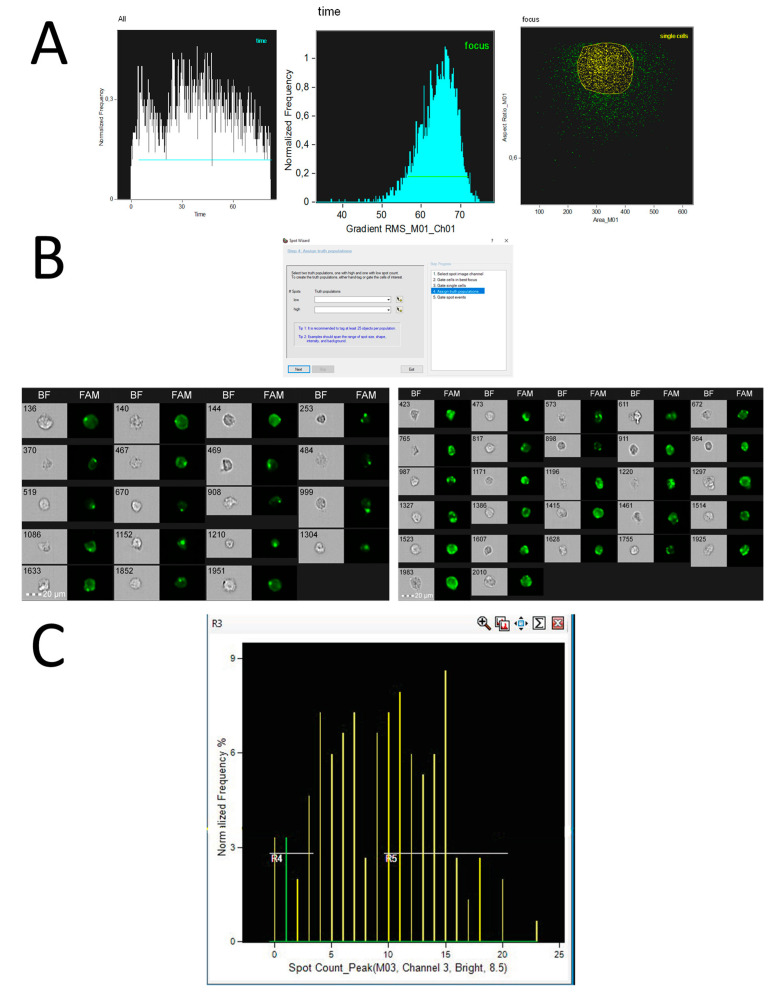
The detection of hybridization spots in MT-4 culture fixed cells using imaging flow cytometry (Cytek^®^ Amnis^®^ Flow Sight) with the Spot Localization wizard. The Spot Localization wizard is an algorithm in the Amnis IDEAS 6.2 software designed to count fluorescence points generated during in situ fluorescence hybridization. In this study, custom probes labeled with FAM (a fluorescent dye) were used to target and bind to specific HIV genes. Fluorescence was excited using a 488 nm laser with a power of 60 mW, and detection was performed in the green channel using a 532/55 nm light filter. The images were captured at a 20× magnification (lens NA = 0.6), with a pixel size of 1 × 1 µm. (**A**)—The gating strategy for isolating single-cells. The analysis begins by gating cells that appear in the camera’s field of view 10–20 s after recording starts. This step helps eliminate potential contamination from the previous sample. Next, cells in focus are selected, followed by the isolation of single-cells for further analysis. (**B**)—Using the “Spot Wizard” tool in IDEAS 6.2 software. The “Spot Wizard” tool was used to separate cells based on the number of hybridization spots. The cells were divided into two populations: those with a low number of hybridization spots (1–3 spots) and those with a high number of hybridization spots (>4 spots). The left plot shows cells with a low number of spots, while the right plot shows cells with a high number of spots. (**C**)—The distribution of hybridization signals. The plot generated by IDEAS 6.2 software shows the distribution of cells based on the number of hybridization signals. The X-axis represents the number of hybridization points detected, while the Y-axis shows the normalized number of cells.

The method for measuring hybridization or fluorescent antibody spots using imaging flow cytometry is shown in Figure 2. The analysis was performed using IDEAS 6.2 software, and the data were recorded in .rif files from the Amnis FlowSight system. This approach allows for the precise quantification of labeled spots in flow cytometry experiments.

Cytek^®^ Amnis^®^ offers software tools for automated analysis, including a built-in assistant for counting fluorescence spots (Spot, Figure 2B). This feature guides users step-by-step to isolate specific cell subpopulations, such as those with different numbers of HIV proviruses, and provides the final count. Additionally, newer versions of IDEAS (Cytek^®^ Amnis^®^ data analysis software) support machine learning methods, which help speed up the analysis process [19,20].

**Figure 3:**
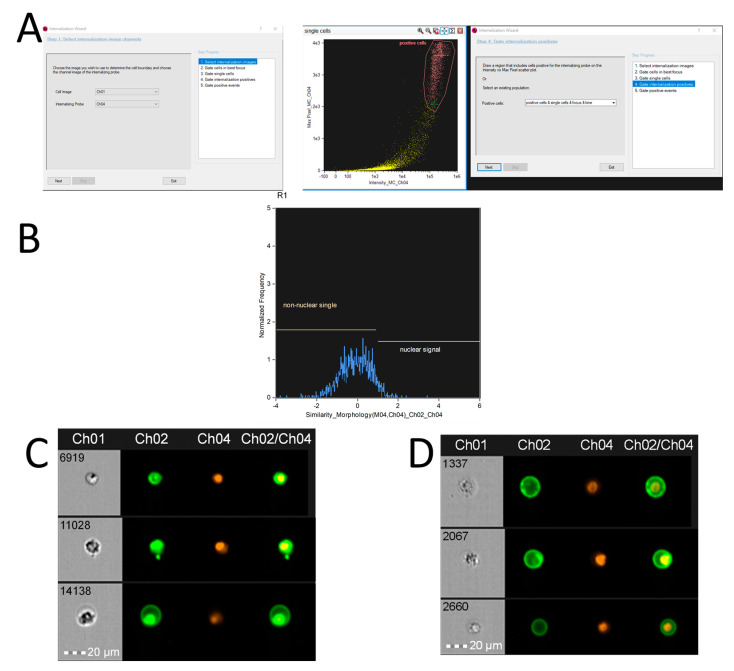
The algorithm for using the Nuclear Localization wizard in IDEAS 6.2 software. The Nuclear Localization wizard is a tool in the IDEAS software designed to determine whether a fluorescent label is located inside the cell (cytoplasm) or outside the cell (nucleus). This tool uses the *Similarity Feature* to compare the correlation between the fluorescent signals in different parts of the cell. For accurate results, two labels are required: one for the cytoplasm and one for the test signal. If the signals show a positive correlation, the test signal is located in the cytoplasm. If the correlation is negative, the signal is localized in the nucleus. The images used in this analysis were obtained from MT4 fixed cells using imaging flow cytometry (Cytek^®^ Amnis^®^ Flow Sight). The cells were stained with Annexin V-FITC and propidium iodide (PI) to assess cell viability. Fluorescence was excited using a 488 nm laser with a power of 60 mW. The green fluorescence from FITC was detected in the second channel using a 532/55 nm filter, while the red fluorescence from PI was detected in the third channel using a 577/35 nm filter. The images were captured at a total magnification of 20× (lens numerical aperture = 0.6), with a pixel size of 1 × 1 µm. (**A**)—The selection of the channel for whole-cell images and the probe channel, identifying cells with positive fluorescent signals. (**B**)—The output of the Nuclear Localization wizard algorithm, showing two distinct cell populations: one with nuclear signal localization (right gated population) and one with extranuclear localization (left gated population). (**C**)—A sample of cell images with nuclear signal localization, identified by the Nuclear Localization wizard algorithm. (**D**)—A sample of cell images with extranuclear signal localization, identified by the Nuclear Localization wizard algorithm.

Another useful tool in IDEAS 6.2 is the Nuclear Localization wizard. This feature helps identify and analyze events with fluorescent signals specifically located within the cell nucleus. When using DNA-DNA FISH, the wizard ensures the isolation of a clean subpopulation of cells by excluding those with background or incorrect staining in the cytoplasm (Figure 3).

## 4. Cellular Cytoskeleton During HIV Infection

The cytoskeleton is a dynamic structure inside cells made up of three main parts: microtubules, intermediate filaments, and actin microfilaments [52]. Actin microfilaments are the smallest of these, with a diameter of 6–8 nm. They help maintain the cell’s shape and can attach to the inner side of the cell membrane, allowing parts of the membrane to move relative to each other. Microtubules, the largest cytoskeletal structures at 25 nm in diameter, are made of two key proteins: alpha and beta tubulin. These structures play a crucial role in moving materials within the cell, using energy from ATP. Transport occurs in both directions, towards the “plus” and “minus” ends of the microtubules. Importantly, this system is also used to transport proteins from the HIV capsid [57].

During HIV infection, the cytoskeleton changes due to both the cell’s antiviral response and the actions of early HIV proteins (Nef, Rev, Tat, and Vif). Actin microfilaments act as a barrier to HIV entry by maintaining the cell membrane’s structure [58]. However, they also help cluster CD4 receptors with co-receptors (CCR5 or CXCR4) on the cell surface, which are essential for HIV to enter the cell. Microtubules are critical for moving HIV proteins and possibly the pre-integration complex (a key viral structure) toward the nucleus. Additionally, the cytoskeleton plays a central role in how HIV interacts with other cells and spreads from cell to cell. This cell-to-cell transmission is thought to be the main way HIV spreads in the body [59].

## 5. Actin Microfilaments

Actin microfilaments are a key part of the cytoskeleton, responsible for maintaining cell shape and enabling cell contraction. They also play a role in forming connections between cells. Importantly, actin microfilaments help concentrate surface receptors in specific areas of the cell, which is critical for HIV entry and its spread from one cell to another [58].

When the HIV protein gp120 binds to the CD4 receptor on a cell, it triggers a series of signals that cause the CD4 receptor and its co-receptors to cluster together. This clustering is driven by actin and helps stabilize the fusion pores, which are essential for HIV to enter the cell [60]. During this process, the cell membrane undergoes structural changes, and HIV moves along the membrane to find the best entry point—a process known as “viral surfing” [61].

The speed of this process depends on how quickly the actin cytoskeleton reorganizes and how fast the CD4 and co-receptors cluster.

**Figure 4:**
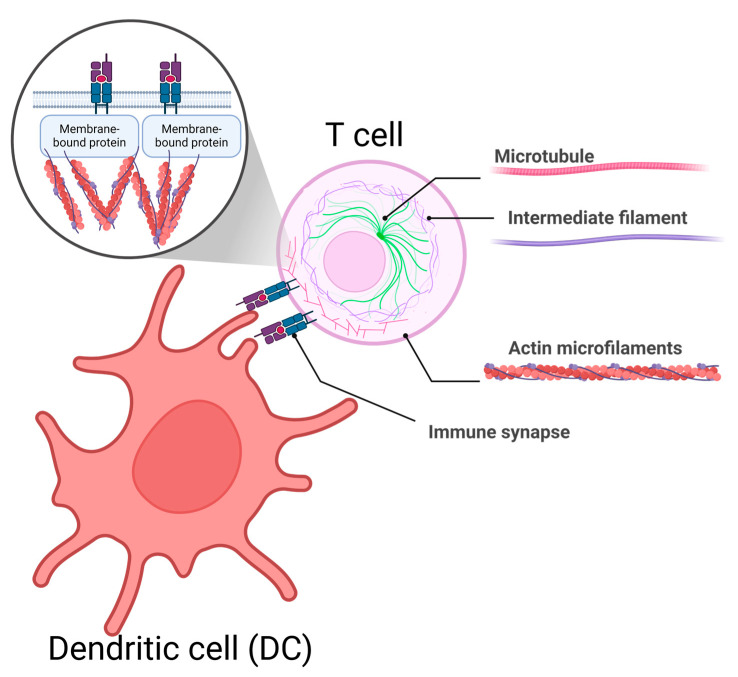
Actin reorganization during the formation of the immunological synapse between a CD4+ cell and an antigen-presenting cell (APC). Actin filaments, which are part of the cell’s cytoskeleton, help concentrate receptors on the cell membrane, enabling interactions between the two cells.

Studying these events using traditional flow cytometry is challenging. While flow cytometry can measure fluorescence, it cannot provide detailed images of what is happening at the cellular level. For example, the total number of receptors and actin microfilaments might stay the same, so the overall fluorescence signal may not change. However, flow cytometry combined with visualization techniques can overcome this limitation. It allows researchers to focus on specific areas of cell interaction and analyze changes in fluorescence intensity in those regions (Figure 4).

HIV also alters actin microfilaments after it enters the cell, particularly when early regulatory HIV proteins are produced. One such protein, Nef, interacts with a host protein called PAK2. The SH3 domain of Nef binds to PAK2, forming a Nef-PAK2 complex [62]. This complex disrupts the formation of F-actin microfilaments at the cell’s edges, reducing the cell’s mobility and its ability to form immunological synapses. However, Nef does not affect F-actin in structures like filopodia and nanotubes, which allows the infected cell to remain mobile and continue spreading HIV to other cells [63].

Imaging flow cytometry is a powerful tool for studying changes in the actin cytoskeleton during HIV infection. In one study, researchers used this method to study how the HIV-1 Nef protein affects the actin cytoskeleton in infected cells. By visualizing and measuring changes in actin dynamics, they observed that Nef inhibits the formation of membrane folds and filopodial protrusions in lymphocytes [64]. To analyze these changes, the researchers first selected single-cells based on bright-field images using parameters such as area and Aspect Ratio. They then focused on cells in clear view using the gradient root mean square (RMS) parameter. The nucleus of each cell was identified using a DAPI channel mask. To exclude apoptotic cells, a Threshold mask (40%) was applied to the area feature. Finally, round cells and cells with filopodia (formed by actin microfilaments) were distinguished using features like Bright Detail Intensity R3, Symmetry 3, Circularity, and a Morphology mask.

In another study, imaging flow cytometry was used to examine actin polymerization at the immunological synapse—the contact point between dendritic cells and conventional CD4+ T cells. This approach helped quantify how regulatory T cells influence actin dynamics, which is essential for proper synapse formation and viral transmission [65]. The method was particularly valuable because it allowed for the analysis of a large number of events while providing detailed information about the spatial distribution of fluorescent signals. Using this technique, the researchers identified immune synapse events among cells positive for CFSE and HLA-DR markers.

**Figure 5:**
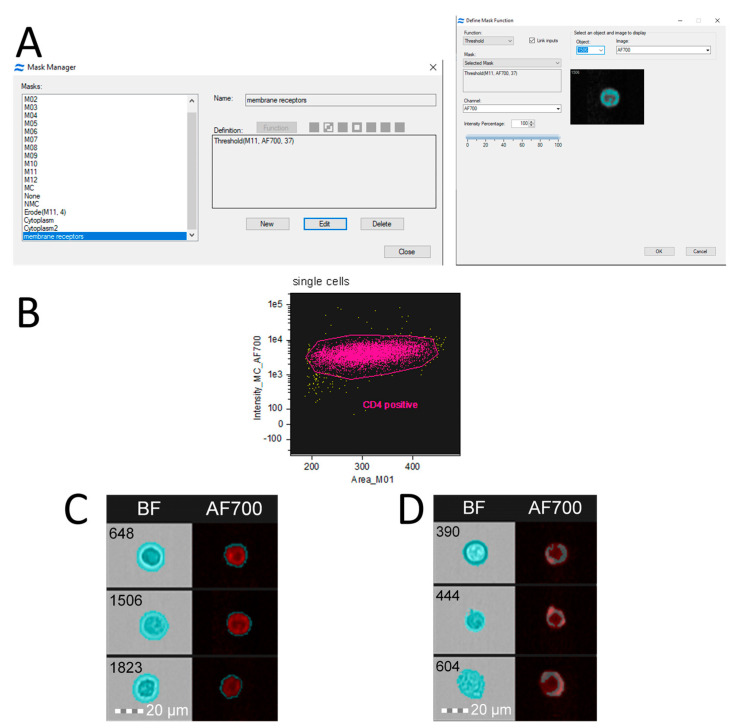
Visualization of CD4 receptor localization on the cytoplasmic membrane using IDEAS 6.2 software. The CD4 receptors were labeled with Alexa Fluor 700 (AF700) antibodies (BD Biosciences, Becton, Dickinson and Company, Franklin Lakes, New Jersey, USA). The images were captured from MT4 cell cultures using imaging flow cytometry (Cytek^®^ Amnis^®^ FlowSight). A 642 nm laser with 100 mW power was used to excite the fluorescence, which was detected in the red channel using a 702/85 nm filter for AF700. The images were taken at 20× magnification (lens NA = 0.6), with a pixel size of 1 × 1 µm. (**A**)—The creation of a simple mask to isolate the membrane stained with AF700-labeled antibodies against CD4. The Threshold parameter was set to exclude pixels with brightness below 37. (**B**)—A graph showing the relationship between cell area and fluorescence intensity of AF700-labeled CD4 receptors. (**C**)—The bright-field and fluorescent images of the cells with uniform CD4 receptor distribution on the membrane. The left image shows the bright-field view with a LevelSet mask, while the right image displays the fluorescent signal with a custom membrane receptor mask applied. (**D**)—The images of the cells with uneven CD4 receptor distribution on the cytoplasmic membrane.

Certain software tools, such as IDEAS 6.2 (Figure 5), can identify specific areas of a cell’s outer membrane and measure fluorescence levels in those regions. To clearly visualize the distribution of CD4 receptors on the membrane, a specialized mask can be generated. This mask is created by focusing on areas with the highest fluorescence signals from antibodies that bind to the CD4 receptors (using the “Peak” parameter) and then subtracting the general cell outline (using the “Morphology” parameter). The resulting mask, called the “receptor localization” mask, highlights where CD4 receptor clusters are located. As illustrated in Figure 5, CD4 receptors can either be spread evenly across the membrane (Figure 5C) or grouped into distinct clusters (Figure 5D). These clusters play a key role in enabling the transmission of HIV [66].

**Figure 6:**
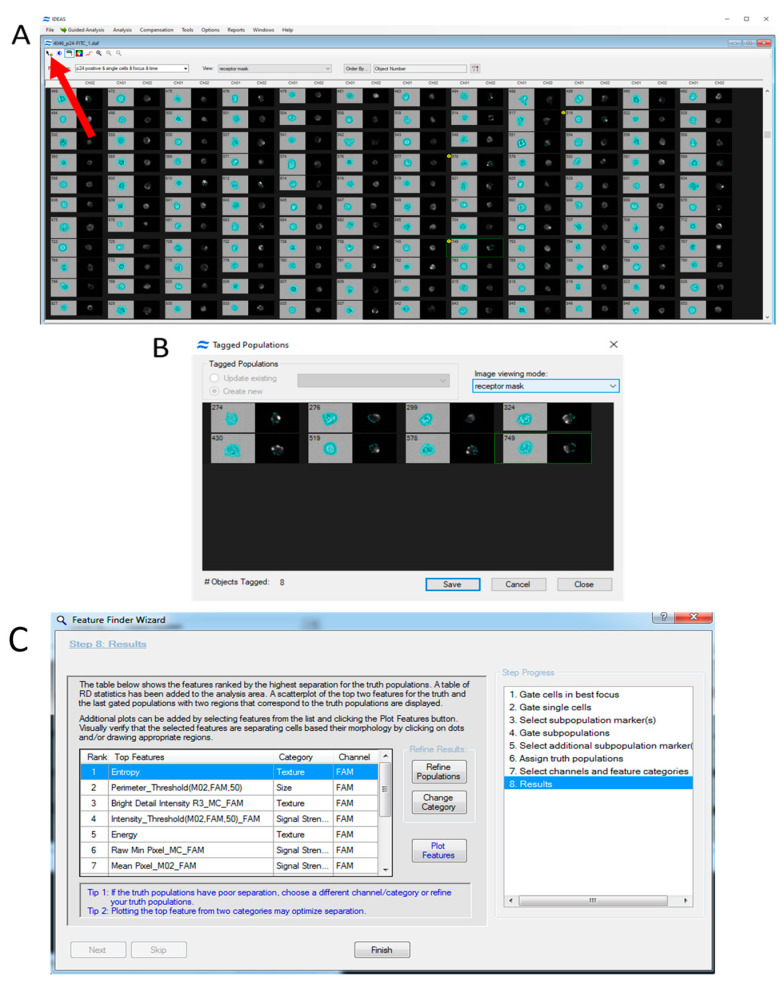
The selection of individual events in the IDEAS 6.2 software image gallery for further analysis of cell subpopulations. (**A**)—Switching to the manual event selection mode. (**B**)—The images of user-selected events, in this case, cells with an uneven distribution of CD4 receptors on the cell membrane. (**C**)—The results from the Feature Finder Wizard algorithm in IDEAS. Features showing the largest differences between subpopulations are ranked from highest to lowest. The analysis revealed differences in entropy (a measure of how evenly the fluorescent signal is distributed) and pixel brightness (Bright Detail Intensity) between cell subpopulations with 1–3 and more than 4 hybridization points of probes targeting the HIV genome.

When analyzing specific events of interest, these events can be separated into distinct subpopulations for further study (Figure 6). Instead of manually sorting cells, an automated approach can be used to divide cells into subpopulations. For example, the Feature Finder Wizard algorithm can be applied. This tool requires the selection of fluorescence channels and the identification of the populations to be analyzed. The algorithm then generates a list of morphological parameters that show the most significant differences between the selected populations.

## 6. Direct Detection of HIV Gene Expression and HIV Protein Interactions with Host Cell Proteins in Same Cell Compartments

Molecular cloning and genetic engineering techniques allow researchers to create reporter gene systems. These systems can be used to study the expression of HIV genes [67] or to modify gene sequences so that HIV proteins can be visualized using fluorescent markers [68,69].

A reporter gene is a gene that produces a fluorescent protein and is linked to the promoter of another gene [70]. When the promoter is activated, the fluorescent protein is produced along with the HIV gene or genes, making it possible to track their expression. This approach has several benefits: it avoids the need for additional staining or complex preparation steps, provides quick and consistent results, and allows researchers to study gene expression in living cells.

Talia H. Swartz et al. conducted a study to examine how cell receptors (CXCR4) bind to viral particles [71]. The researchers used a modified version of HIV-1, which included a reporter gene to track the expression of the HIV Gag protein.

Label co-localization studies can also be applied to standard immunocytochemistry and FISH techniques without requiring genetic constructs with reporter genes. For example, Kiera L. Clayton et al. used a co-localization tool to analyze the presence of HIV Env and Gag proteins, as well as host cell proteins EEA1, LAMP1, and Siglec-1, within specific cellular compartments [72]. By comparing co-localization patterns in HIV-infected macrophages to uninfected control cells, the study provided insights into how HIV infection alters cellular processes, particularly in relation to immune evasion strategies used by infected macrophages.

Filippos Poruchik et al. developed a new FISH method using probes with LNA modifications to detect specific mRNAs (IFNγ, IL-2) and microRNAs (miR-155) [73]. Their work also explored the localization of labels for CD3 and CD4 receptors, as well as IFNγ protein and mRNA.

The Cytek^®^ Amnis^®^ imaging flow cytometers use the IDEAS software, which includes a specialized tool called the Co-localization Wizard. This tool helps analyze signals from stained proteins by showing how two dyes overlap in cells. The wizard guides users step-by-step through the analysis process. First, it asks to select the fluorescence channels used in the experiment. Next, it helps identify focused and single-cells. Finally, it allows the selection of cells that show fluorescence. The result of the wizard’s function is the identification of a population of cells where the two proteins, labeled with fluorescent dyes, are found together.

## 7. Conclusions

Imaging flow cytometry is a powerful technique that combines the strengths of two key methods in cell biology: flow cytometry and microscopy. It allows researchers to analyze a large number of cells (providing strong statistical data) while also capturing detailed images of each cell. This dual capability makes it a valuable tool for studying complex biological processes. A key feature of imaging flow cytometry is the use of masks during image processing. Masks help to enhance the visualization and contrast of specific cell components, making it easier to study individual parts of a cell.

Additionally, imaging flow cytometry platforms come with advanced software that can identify and separate different cell compartments or sort cells into subpopulations for further analysis.

These features make imaging flow cytometry particularly useful for studying HIV infection. HIV has a complex life cycle and interacts with host cells through various regulatory proteins. Understanding how HIV affects cell metabolism and spreads from cell to cell remains an important area of research. Imaging flow cytometry provides a way to explore these processes in detail.

The main types of masks used in imaging flow cytometry for HIV research are summarized in Table 1.

Imaging flow cytometry has some limitations compared to traditional methods. For example, it records events at a slower rate and currently does not support cell sorting. Additionally, it may lack the resolution and three-dimensional imaging capabilities found in HCI.

On the other hand, HCI offers several advantages, including high throughput, automation through convolutional neural networks, detailed analysis of cellular features, and the ability to capture three-dimensional images (X, Y, and Z planes). Imaging flow cytometry, however, excels in tasks like cell classification, morphological analysis, and detecting rare events. Both methods have unique strengths and can complement each other in laboratory settings.

While imaging flow cytometry is not a full replacement for other tools, ongoing technological advancements may lead to improved versions. Future instruments could integrate cell sorting and enhanced resolution, driven by innovations in electronics and optics.

## Figures and Tables

**Table 1:** Tools for image analysis in imaging flow cytometry used to study specific cellular processes during HIV infection.

Research Subject	Research Method	Imaging in IDEAS 6.2	References
Latent HIV reservoirs	FISH	Spot counting wizard; Nuclear Localization wizard	[55,56]
Gene transcripts	FISH	Spot counting wizard	[73]
Clustering of cellular receptors	Immunocytochemistry	Peak mask	[65,66]
Cytoskeletal remodeling	Immunocytochemistry	Thershold mask Interface mask	[64]
Detection of proteins in single-cell compartments	Immunocytochemistry	Co-localization mask	[71,72]

## Data Availability

No new data were created or analyzed in this study. Data sharing is not applicable to this article.
